# 2-Cyano­quinolin-1-ium hydrogen sulfate

**DOI:** 10.1107/S160053681003878X

**Published:** 2010-10-02

**Authors:** Wan-Sin Loh, Madhukar Hemamalini, Hoong-Kun Fun

**Affiliations:** a X-ray Crystallography Unit, School of Physics, Universiti Sains Malaysia, 11800 USM, Penang, Malaysia

## Abstract

The title salt, C_10_H_7_N_2_^+^·HSO_4_^−^, is formed by the transfer of a proton from H_2_SO_4_ to the N atom of 2-cyano­quinoline during crystallization. The quinoline ring system is approximately planar with a maximum deviation of 0.013 (3) Å. In the crystal, the cations are linked to the anions *via* inter­molecular N—H⋯O, O—H⋯O and C—H⋯O hydrogen bonds, forming a layered network.

## Related literature

For background to and the biological activity of quinoline derivatives, see: Loh *et al.* (2010*a* ▶ ▶,*b*); Sasaki *et al.* (1998 ▶); Reux *et al.* (2009 ▶); Morimoto *et al.* (1991 ▶); Michael (1997 ▶); Markees *et al.* (1970 ▶); Campbell *et al.* (1988 ▶). For related structures, see: Loh *et al.* (2010*a* ▶,*b* ▶). For the stability of the temperature controller used in the data collection, see: Cosier & Glazer (1986 ▶). For bond-length data, see: Allen *et al.* (1987 ▶).
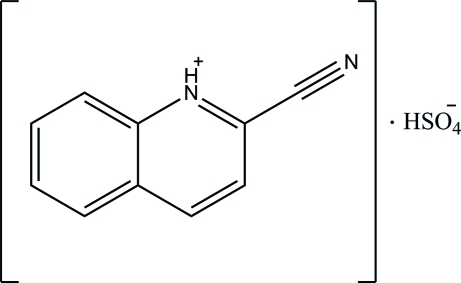


## Experimental

### 

#### Crystal data

C_10_H_7_N_2_^+^·HSO_4_^−^
*M* *_r_* = 252.24
Triclinic, 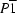

*a* = 7.2154 (3) Å
*b* = 8.2334 (4) Å
*c* = 9.9985 (4) Å
α = 110.622 (2)°
β = 90.982 (3)°
γ = 110.791 (2)°
*V* = 512.82 (4) Å^3^
*Z* = 2
Mo *K*α radiation
μ = 0.32 mm^−1^
*T* = 100 K
0.34 × 0.19 × 0.12 mm

#### Data collection

Bruker SMART APEXII CCD diffractometer
Absorption correction: multi-scan (*SADABS*; Bruker, 2009 ▶) *T*_min_ = 0.900, *T*_max_ = 0.963
5740 measured reflections
1979 independent reflections
1721 reflections with *I* > 2σ(*I*)
*R*_int_ = 0.032

#### Refinement

*R*[*F* ^2^ > 2σ(*F* ^2^)] = 0.051
*wR*(*F* ^2^) = 0.154
*S* = 1.11
1979 reflections
186 parameters
All H-atom parameters refined
Δρ_max_ = 0.82 e Å^−3^
Δρ_min_ = −0.56 e Å^−3^

### 

Data collection: *APEX2* (Bruker, 2009 ▶); cell refinement: *SAINT* (Bruker, 2009 ▶); data reduction: *SAINT*; program(s) used to solve structure: *SHELXTL* (Sheldrick, 2008 ▶); program(s) used to refine structure: *SHELXTL*; molecular graphics: *SHELXTL*; software used to prepare material for publication: *SHELXTL* and *PLATON* (Spek, 2009 ▶).

## Supplementary Material

Crystal structure: contains datablocks global, I. DOI: 10.1107/S160053681003878X/hb5657sup1.cif

Structure factors: contains datablocks I. DOI: 10.1107/S160053681003878X/hb5657Isup2.hkl

Additional supplementary materials:  crystallographic information; 3D view; checkCIF report

## Figures and Tables

**Table 1:** Hydrogen-bond geometry (Å, °)

*D*—H⋯*A*	*D*—H	H⋯*A*	*D*⋯*A*	*D*—H⋯*A*
N1—H1*N*1⋯O1^i^	0.98 (5)	1.71 (5)	2.669 (3)	169 (4)
O4—H1*O*4⋯O2^ii^	0.67 (5)	1.97 (5)	2.641 (3)	176 (7)
C2—H2*A*⋯O2^iii^	0.91 (4)	2.53 (4)	3.320 (4)	145 (3)
C5—H5*A*⋯O4^iv^	0.98 (3)	2.52 (3)	3.475 (4)	166 (3)
C7—H7*A*⋯O3^iv^	0.94 (4)	2.41 (4)	3.338 (4)	167 (3)
C8—H8*A*⋯O2^v^	0.99 (4)	2.59 (4)	3.309 (4)	130 (3)

Symmetry codes: (i) 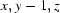; (ii) 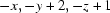; (iii) 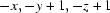; (iv) 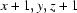; (v) 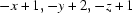.

**Fig. 1.**
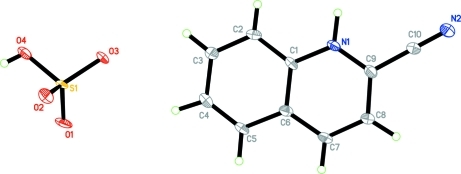
The molecular structure of (I) showing 50% probability displacement ellipsoids for non-H atoms.

**Fig. 2.**
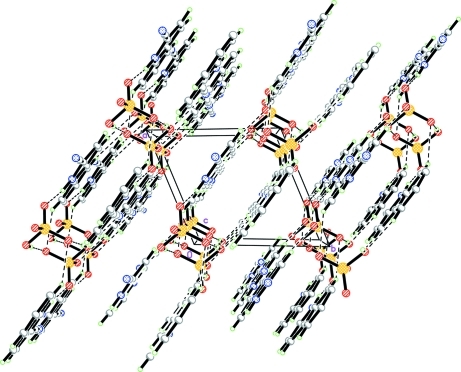
The crystal structure of (I), viewed along the c axis.

## supplementary crystallographic information

### Comment

Recently, hydrogen-bonding patterns involving quinoline and its derivatives with organic acid have been investigated (Loh *at al.*, 2010*a,b*). Syntheses of the quinoline derivatives were discussed earlier (Sasaki *et al.*, 1998; Reux *et al.*, 2009). Quinolines and their derivatives are very important compounds because of their wide occurrence in natural products (Morimoto *et al.*, 1991; Michael, 1997) and biologically active compounds (Markees *et al.*, 1970; Campbell *et al.*, 1988). Heterocyclic molecules containing cyano group are useful as drug intermediates. Herein we report the synthesis of 2-cyanoquinolin-1-ium hydrogen sulfate.

The asymmetric unit of the title compound (Fig. 1) consists of one 2-cyanoquinolin-1-ium cation (C1–C10/N1/N2) and one hydrogen sulfate anion (O1–O4/S1). One proton is transferred from the hydroxyl group of hydrogen sulfate to the atom N1 of 2-cyanoquinoline during the crystallization, resulting in the formation of salt. The quinoline ring system (C1–C9/N1) is approximately planar with a maximum deviation of 0.013 (3) Å at atom C6. Bond lengths (Allen *et al.*, 1987) and angles are within the normal ranges and are comparable to the related structures (Loh *et al.*, 2010**a*,b*).

In the crystal (Fig. 2), the cations are linked by the anions *via* intermolecular N1—H1N1···O1, O4—H1O4···O2, C2—H2A···O2, C5—H5A···O4, C7—H7A···O3 and C8—H8A···O2 hydrogen bonds (Table 1) into a two-dimensional networks.

### Experimental

A few drops of sulfuric acid were added to a hot methanol solution (20 ml) of quinoline-2-carbonitrile (39 mg, Aldrich) which had been warmed over a magnetic stirrer hotplate for a few minutes. The resulting solution was allowed to cool slowly to room temperature. Colourless blocks of (I) appeared after a few days.

### Refinement

All H atoms were located from a difference Fourier map and refined freely with the bond lengths of C–H being 0.91 (3) to 0.99 (3) Å, N–H being 0.98 (5) Å and O–H being 0.67 (5) Å.

### Crystal data

**Table:**

C_10_H_7_N_2_^+^·HSO_4_^−^	*Z* = 2
*M**_r_* = 252.24	*F*(000) = 260
Triclinic, *P*1	*D*_x_ = 1.634 Mg m^−^^3^
Hall symbol: -P 1	Mo *K*α radiation, λ = 0.71073 Å
*a* = 7.2154 (3) Å	Cell parameters from 2998 reflections
*b* = 8.2334 (4) Å	θ = 2.2–27.6°
*c* = 9.9985 (4) Å	µ = 0.32 mm^−^^1^
α = 110.622 (2)°	*T* = 100 K
β = 90.982 (3)°	Block, colourless
γ = 110.791 (2)°	0.34 × 0.19 × 0.12 mm
*V* = 512.82 (4) Å^3^	

### Data collection

**Table:**

Bruker SMART APEXII CCD diffractometer	1979 independent reflections
Radiation source: fine-focus sealed tube	1721 reflections with *I* > 2σ(*I*)
graphite	*R*_int_ = 0.032
φ and ω scans	θ_max_ = 26.0°, θ_min_ = 2.2°
Absorption correction: multi-scan (*SADABS*; Bruker, 2009)	*h* = −8→6
*T*_min_ = 0.900, *T*_max_ = 0.963	*k* = −10→10
5740 measured reflections	*l* = −12→12

### Refinement

**Table:**

Refinement on *F*^2^	Primary atom site location: structure-invariant direct methods
Least-squares matrix: full	Secondary atom site location: difference Fourier map
*R*[*F*^2^ > 2σ(*F*^2^)] = 0.051	Hydrogen site location: inferred from neighbouring sites
*wR*(*F*^2^) = 0.154	All H-atom parameters refined
*S* = 1.11	*w* = 1/[σ^2^(*F*_o_^2^) + (0.0884*P*)^2^ + 0.5522*P*] where *P* = (*F*_o_^2^ + 2*F*_c_^2^)/3
1979 reflections	(Δ/σ)_max_ < 0.001
186 parameters	Δρ_max_ = 0.82 e Å^−^^3^
0 restraints	Δρ_min_ = −0.56 e Å^−^^3^

### Special details

**Table:**

Experimental. The crystal was placed in the cold stream of an Oxford Cryosystems Cobra open-flow nitrogen cryostat (Cosier & Glazer, 1986) operating at 100.0 (1) K.
Geometry. All e.s.d.'s (except the e.s.d. in the dihedral angle between two l.s. planes) are estimated using the full covariance matrix. The cell e.s.d.'s are taken into account individually in the estimation of e.s.d.'s in distances, angles and torsion angles; correlations between e.s.d.'s in cell parameters are only used when they are defined by crystal symmetry. An approximate (isotropic) treatment of cell e.s.d.'s is used for estimating e.s.d.'s involving l.s. planes.
Refinement. Refinement of *F*^2^ against ALL reflections. The weighted *R*-factor *wR* and goodness of fit *S* are based on *F*^2^, conventional *R*-factors *R* are based on *F*, with *F* set to zero for negative *F*^2^. The threshold expression of *F*^2^ > σ(*F*^2^) is used only for calculating *R*-factors(gt) *etc*. and is not relevant to the choice of reflections for refinement. *R*-factors based on *F*^2^ are statistically about twice as large as those based on *F*, and *R*- factors based on ALL data will be even larger.

### Fractional atomic coordinates and isotropic or equivalent isotropic displacement parameters (Å^2^)

**Table:**

	*x*	*y*	*z*	*U*_iso_*/*U*_eq_	
S1	0.15454 (10)	0.99553 (10)	0.33365 (7)	0.0141 (3)	
O1	0.3450 (3)	1.0495 (3)	0.4227 (2)	0.0216 (5)	
O2	0.0561 (3)	1.1258 (3)	0.3966 (2)	0.0184 (5)	
O3	0.1678 (3)	0.9584 (3)	0.1832 (2)	0.0199 (5)	
O4	0.0149 (3)	0.8001 (3)	0.3345 (3)	0.0213 (5)	
N1	0.5709 (4)	0.3044 (3)	0.6749 (3)	0.0153 (5)	
N2	0.7421 (5)	0.4421 (4)	0.4025 (3)	0.0341 (8)	
C1	0.5498 (4)	0.2853 (4)	0.8046 (3)	0.0146 (6)	
C2	0.3903 (4)	0.1311 (4)	0.8119 (3)	0.0171 (6)	
C3	0.3736 (5)	0.1152 (4)	0.9439 (3)	0.0176 (6)	
C4	0.5161 (4)	0.2496 (4)	1.0697 (3)	0.0178 (7)	
C5	0.6693 (4)	0.3999 (4)	1.0632 (3)	0.0157 (6)	
C6	0.6915 (4)	0.4236 (4)	0.9298 (3)	0.0151 (6)	
C7	0.8438 (4)	0.5775 (4)	0.9151 (3)	0.0161 (6)	
C8	0.8587 (4)	0.5909 (4)	0.7818 (3)	0.0164 (6)	
C9	0.7188 (4)	0.4485 (4)	0.6622 (3)	0.0171 (6)	
C10	0.7297 (5)	0.4459 (4)	0.5172 (3)	0.0224 (7)	
H2A	0.305 (5)	0.040 (5)	0.730 (4)	0.017 (8)*	
H3A	0.266 (5)	0.008 (5)	0.947 (4)	0.018 (8)*	
H4A	0.493 (5)	0.226 (5)	1.157 (4)	0.025 (9)*	
H5A	0.767 (4)	0.498 (4)	1.148 (3)	0.008 (7)*	
H7A	0.933 (5)	0.672 (5)	0.999 (4)	0.016 (8)*	
H8A	0.964 (5)	0.696 (5)	0.767 (4)	0.015 (8)*	
H1N1	0.474 (6)	0.211 (6)	0.589 (5)	0.045 (11)*	
H1O4	−0.005 (7)	0.814 (7)	0.402 (5)	0.040 (14)*	

### Atomic displacement parameters (Å^2^)

**Table:**

	*U*^11^	*U*^22^	*U*^33^	*U*^12^	*U*^13^	*U*^23^
S1	0.0155 (4)	0.0150 (4)	0.0056 (4)	0.0016 (3)	−0.0029 (3)	0.0015 (3)
O1	0.0213 (11)	0.0246 (12)	0.0092 (10)	0.0065 (9)	−0.0058 (8)	−0.0016 (9)
O2	0.0221 (11)	0.0209 (11)	0.0133 (10)	0.0093 (9)	0.0018 (8)	0.0069 (9)
O3	0.0229 (11)	0.0231 (12)	0.0058 (10)	0.0025 (9)	0.0009 (8)	0.0031 (9)
O4	0.0286 (13)	0.0204 (12)	0.0079 (11)	0.0045 (10)	0.0005 (9)	0.0028 (10)
N1	0.0176 (13)	0.0158 (13)	0.0088 (12)	0.0046 (10)	−0.0020 (10)	0.0025 (10)
N2	0.0415 (18)	0.0289 (16)	0.0134 (14)	−0.0059 (14)	−0.0036 (12)	0.0070 (12)
C1	0.0183 (15)	0.0194 (15)	0.0080 (13)	0.0100 (12)	0.0014 (11)	0.0047 (12)
C2	0.0155 (14)	0.0167 (15)	0.0130 (15)	0.0041 (12)	−0.0030 (12)	0.0013 (12)
C3	0.0175 (15)	0.0185 (16)	0.0148 (15)	0.0047 (13)	−0.0003 (12)	0.0066 (13)
C4	0.0218 (16)	0.0229 (16)	0.0113 (15)	0.0116 (13)	0.0031 (12)	0.0063 (13)
C5	0.0183 (15)	0.0199 (15)	0.0079 (14)	0.0094 (13)	−0.0005 (11)	0.0023 (12)
C6	0.0159 (14)	0.0145 (15)	0.0122 (14)	0.0051 (12)	−0.0019 (11)	0.0030 (12)
C7	0.0197 (15)	0.0157 (15)	0.0093 (14)	0.0073 (12)	−0.0016 (12)	0.0004 (12)
C8	0.0154 (14)	0.0162 (15)	0.0134 (14)	0.0033 (12)	−0.0013 (11)	0.0039 (12)
C9	0.0209 (15)	0.0182 (15)	0.0113 (14)	0.0069 (12)	0.0007 (11)	0.0055 (12)
C10	0.0259 (17)	0.0183 (16)	0.0134 (16)	0.0001 (13)	−0.0026 (12)	0.0040 (13)

### Geometric parameters (Å, °)

**Table:**

S1—O3	1.438 (2)	C3—C4	1.425 (4)
S1—O1	1.457 (2)	C3—H3A	0.96 (3)
S1—O2	1.460 (2)	C4—C5	1.358 (4)
S1—O4	1.570 (2)	C4—H4A	0.96 (4)
O4—H1O4	0.67 (5)	C5—C6	1.418 (4)
N1—C9	1.333 (4)	C5—H5A	0.98 (3)
N1—C1	1.365 (4)	C6—C7	1.408 (4)
N1—H1N1	0.98 (5)	C7—C8	1.378 (4)
N2—C10	1.142 (4)	C7—H7A	0.95 (4)
C1—C2	1.404 (4)	C8—C9	1.398 (4)
C1—C6	1.426 (4)	C8—H8A	0.99 (3)
C2—C3	1.375 (4)	C9—C10	1.446 (4)
C2—H2A	0.91 (3)		
			
O3—S1—O1	113.75 (13)	C5—C4—C3	120.9 (3)
O3—S1—O2	113.56 (12)	C5—C4—H4A	124 (2)
O1—S1—O2	111.77 (12)	C3—C4—H4A	115 (2)
O3—S1—O4	104.10 (13)	C4—C5—C6	120.2 (3)
O1—S1—O4	105.98 (13)	C4—C5—H5A	123.1 (17)
O2—S1—O4	106.81 (13)	C6—C5—H5A	116.7 (17)
S1—O4—H1O4	108 (4)	C7—C6—C5	123.5 (3)
C9—N1—C1	122.0 (2)	C7—C6—C1	118.5 (3)
C9—N1—H1N1	119 (3)	C5—C6—C1	118.0 (3)
C1—N1—H1N1	119 (3)	C8—C7—C6	120.6 (3)
N1—C1—C2	119.6 (3)	C8—C7—H7A	121 (2)
N1—C1—C6	118.8 (3)	C6—C7—H7A	118 (2)
C2—C1—C6	121.7 (3)	C7—C8—C9	118.4 (3)
C3—C2—C1	118.2 (3)	C7—C8—H8A	122.8 (19)
C3—C2—H2A	121 (2)	C9—C8—H8A	118.8 (19)
C1—C2—H2A	121 (2)	N1—C9—C8	121.7 (3)
C2—C3—C4	121.0 (3)	N1—C9—C10	116.2 (3)
C2—C3—H3A	117 (2)	C8—C9—C10	122.1 (3)
C4—C3—H3A	121 (2)	N2—C10—C9	178.2 (4)
			
C9—N1—C1—C2	179.6 (3)	C2—C1—C6—C7	−178.0 (3)
C9—N1—C1—C6	−0.5 (4)	N1—C1—C6—C5	−179.0 (2)
N1—C1—C2—C3	179.7 (3)	C2—C1—C6—C5	1.0 (4)
C6—C1—C2—C3	−0.3 (4)	C5—C6—C7—C8	179.2 (3)
C1—C2—C3—C4	−1.1 (5)	C1—C6—C7—C8	−1.9 (4)
C2—C3—C4—C5	1.8 (5)	C6—C7—C8—C9	0.2 (4)
C3—C4—C5—C6	−1.1 (4)	C1—N1—C9—C8	−1.4 (4)
C4—C5—C6—C7	178.7 (3)	C1—N1—C9—C10	176.8 (3)
C4—C5—C6—C1	−0.3 (4)	C7—C8—C9—N1	1.5 (5)
N1—C1—C6—C7	2.0 (4)	C7—C8—C9—C10	−176.6 (3)

### Hydrogen-bond geometry (Å, °)

**Table:**

*D*—H···*A*	*D*—H	H···*A*	*D*···*A*	*D*—H···*A*
N1—H1N1···O1^i^	0.98 (5)	1.71 (5)	2.669 (3)	169 (4)
O4—H1O4···O2^ii^	0.67 (5)	1.97 (5)	2.641 (3)	176 (7)
C2—H2A···O2^iii^	0.91 (4)	2.53 (4)	3.320 (4)	145 (3)
C5—H5A···O4^iv^	0.98 (3)	2.52 (3)	3.475 (4)	166 (3)
C7—H7A···O3^iv^	0.94 (4)	2.41 (4)	3.338 (4)	167 (3)
C8—H8A···O2^v^	0.99 (4)	2.59 (4)	3.309 (4)	130 (3)

Symmetry codes: (i) *x*, *y*−1, *z*; (ii) −*x*, −*y*+2, −*z*+1; (iii) −*x*, −*y*+1, −*z*+1; (iv) *x*+1, *y*, *z*+1; (v) −*x*+1, −*y*+2, −*z*+1.
